# Serum catestatin levels in patients with rheumatoid arthritis

**DOI:** 10.1038/s41598-022-07735-x

**Published:** 2022-03-09

**Authors:** Petra Simac, Dijana Perkovic, Ivona Bozic, Marijana Matijas, Katarina Gugo, Dinko Martinovic, Josko Bozic

**Affiliations:** 1 Division of Clinical Immunology and Rheumatology, Department of Internal Medicine, University Hospital of Split, Split, Croatia; 2 Department of Medical Laboratory Diagnostics, University Hospital of Split, 21000 Split, Croatia; 3 Department of Pathophysiology, School of Medicine, University of Split, 21000 Split, Croatia

**Keywords:** Osteoimmunology, Rheumatic diseases

## Abstract

Catestatin (CST) is an important peptide that influences various inflammatory diseases. Our goal was to investigate CST concentrations in patients with RA compared to healthy subjects. This cross-sectional observational study included 80 patients with RA and 80 healthy control subjects. Demographic characteristics and laboratory parameters were recorded. Serum CST levels were determined by an enzyme-linked immunosorbent assay (ELISA). Serum CST levels were significantly higher in RA patients than in the control group (10.53 ± 3.90 vs 5.24 ± 2.37 ng/mL, p < 0.001). In RA patients, there was a statistically significant correlation between CST and patient age (r = 0.418, p < 0.001) and both DAS28 (r = 0.469, p < 0.001) and HAQ scores (r = 0.483, p < 0.001). There was a statistically significant correlation between serum CST levels and RA duration (r = 0.583, p < 0.001). Multiple linear regression analysis showed that serum CST levels retained a significant association with RA duration (β ± SE, 0.13 ± 0.04, p = 0.002) and DAS28 score (0.94 ± 0.45, p = 0.039) after model adjustment for age, body mass index (BMI) and HAQ score, with serum CST levels as a dependent variable. These findings imply that CST is possibly associated with RA complex pathophysiology and disease activity. However, future larger multicentric longitudinal studies are necessary to define the role of CST in RA.

## Introduction

Rheumatoid arthritis (RA) is a chronic systemic inflammatory disease of unknown etiology that affects 0.5 to 1% of the general population^1^. RA is characterized by progressive joint damage leading to physical disability and is associated with various extra-articular manifestations. It is considered to be caused by a combination of genetic and epigenetic components, abnormalities in the cellular and humoral immune response and exposure to environmental factors (especially cigarette smoke and dust exposure)^2^. Chronic systemic inflammation in RA has a key role in accelerated atherogenesis through direct and indirect effects on the vascular endothelium and myocardium, contributing to higher cardiovascular (CV) risk in addition to classical CV risk factors such as obesity, smoking, dyslipidemia and hypertension^3–6^.

Catestatin (CST) is a multifunctional bioactive peptide that is proteolytically cleaved from chromogranin A (ChgA). It primarily acts as an inhibitor of catecholamine secretion and as a stimulator of histamine release^7–10^. According to previous studies, CST has a significant role in the reduction of endothelial inflammation^7^, regulation of blood pressure^11,12^, suppression of atherosclerosis and inhibition of coronary vasoconstriction^13^. CST is believed to have an important role in the pathophysiology of heart failure, acting as an attenuator of the cardiac inflammation through shifting differentiation of macrophages to more anti-inflammatory phenotypes^14,15^. Moreover, CST is particularly of interest because of its immunomodulatory role, including metabolic regulation and immune homeostasis^10,13,16^. Recently published studies demonstrated significantly increased serum CST levels in patients with inflammatory bowel disease (IBD), which is in concordance with in vitro findings of the impact of CST on intestinal microbiota^17–19^. Those findings have implicated a possible contribution of CST to higher CV risk, as well as its role in anti-inflammatory responses in these patients^18,19^. Interestingly, it was proposed that circulating CST in human plasma could be intermediate hereditary phenotype for increased CV risk^20^.

Previous studies demonstrated that the high prevalence of classical CV risk factors does not fully explain the 50% increased risk of CV-related morbidity and mortality in RA patients^21,22^. However, elevated serum levels of proinflammatory cytokines, tumor necrosis factor alpha (TNF-α), interleukins-1 and -6 (IL-1, IL-6), and autoantibodies contribute to systemic inflammation, which makes RA a major independent CV risk factor^23,24^. Therefore, management of both traditional risk factors and RA disease activity should be imperative^22^. Moreover, recent studies have implicated a potential clinical application of CST and other CgA cleavage products as diagnostic markers, as higher concentrations of these peptides have been detected in patients with CV diseases (CVDs), IBD, chronic kidney disease and RA^14,18,19,25,26^. As already mentioned, several recent studies have shown that CTS attenuates the inflammatory response through the downregulation of macrophage differentiation toward the production of proinflammatory cytokines (TNF-α, IL-1, IL-6)^12,27,28^. Therefore, all of the aforementioned evidence points to a possible link between RA and CST through either an immunomodulatory or atherogenic role. To the best of our knowledge, until this point, there have been no published studies that explored the association of serum CST levels with RA.

The aim of this study was to determine serum CST levels in RA patients in comparison with healthy controls and to assess the relationship between CST levels and chronic inflammation, duration and activity of RA, as well as other clinical, biochemical and anthropometric parameters.

## Methods

### Study design

This cross-sectional observational study was conducted at the Department of Internal Medicine, Division of Clinical Immunology and Rheumatology, University Hospital of Split over a period from October 2020 to June 2021.

### Ethics approval

The study was conducted according to the guidelines of the Declaration of Helsinki of 1975, as revised in 2013, and approved by the Ethics Committee of the University Hospital of Split (Class: 500-03/20-01/109; Registration number: 2181-147-01/06/M.S.-20-02). All subjects were informed about the procedures and purpose of the study in a timely manner. Informed consent was obtained from all subjects involved in the study.

### Subjects

This study included 80 patients with RA and 80 healthy control subjects matched by age and sex. One hundred RA patients were screened for inclusion in the study, and 85 patients were eligible for inclusion in the study. Among the participants mentioned, three were excluded due to refusal of further procedures, and two were excluded due to sudden disease worsening. A total of 120 healthy control subjects were screened at the beginning of the study. Among them, twenty participants were excluded due to inability to schedule an appointment and remaining others due to refusal of further procedures. RA diagnosis was made in accordance with the latest 2010 American College of Rheumatology/European League Against Rheumatism Classification Criteria for RA^29^. All participants were above 18 years of age and underwent the full study protocol, except measurement of disease activity [using Disease Activity Score-28 (DAS28) and functional disability [using The Stanford Health Assessment Questionnaire (HAQ)], which were measured only in the patient group^30–32^. Patients with RA were recruited from the outpatient clinic of the Department of Clinical Immunology and Rheumatology, University Hospital of Split, while control subjects were recruited from healthy blood donors, medical staff and healthy volunteers from the local primary health care center.

Inclusion criteria were: disease duration of at least 2 years; age between 18 and 75 years; mandatory treatment with disease-modifying antirheumatic drugs (DMARDs), including biologic DMARDs (bDMARDs) or targeted synthetic DMARDs (tsDMARDs): TNF inhibitors (TNFi’s) which includes adalimumab, certolizumab, etanercept, golimumab or infliximab, B-cell targeted therapies (rituximab), IL-6 inhibitors (sarilumab or tocilizumab) and Janus kinase (JAK) inhibitors (tofacitinib, baricitinib, upadacitinib).

The exclusion criteria were: chronic inflammatory disorders other than RA; diabetes mellitus; pregnancy; breastfeeding; renal impairment [eGFR (estimated glomerular filtration rate) ≤ 60 mL/min/1.73 m^2^]; history of heart failure; respiratory disease; liver disease; active malignant disease; clinically significant drug abuse and consumption of alcohol more than 40 g/day, and long-term exposure to oral glucocorticoid therapy ≥ 10 mg/day.

All control subjects were screened for the presence of systemic autoimmune diseases, as well as any other symptoms suggestive of inflammation of the musculoskeletal system. Furthermore, all potential control group subjects underwent detailed physical examination along with laboratory analysis [the complete blood count, differential blood count and levels of C-reactive protein (CRP)]. We excluded all participants who showed any sign of inflammation in any of these steps.

### Clinical assessment and anthropometric measurements

All participants had a detailed medical history, a physical examination and anthropometric data assessment. Anthropometric measurements included body height, weight and body mass index (BMI). A calibrated medical scale with built-in heights (Seca, Birmingham, UK) was used to measure body mass and height, and BMI was calculated according to the formula = [body weight (kg)]/[height per square (m^2^)]. Arterial blood pressure was measured using a standard mercury sphygmomanometer Riester Big Ben Aneroid [Rudolf Riester GmbH, Jungingen, Germany] with a suitable arm, and values were obtained after a minimum of two measurements. The subjects were seated and relaxed for 10 min before the measurements, with the upper arm at heart level. The mean values of arterial blood pressure was determined after those two measurements. Data on smoking, coffee and alcohol consumption were taken from all study participants. Finally, we checked the medical records of all patients and extracted clinical data relevant for this study.

### Biochemical analysis

Blood samples were collected from all participants after 12 h of overnight fasting from the cubital vein via a polyethylene catheter. Serum samples were centrifuged, and routine laboratory tests were performed the same day. Sample aliquots for CST analyses were stored at − 80 °C for further analysis. All analyses were performed according to standard laboratory protocols by the same experienced biochemist who was blinded to the participants’ group affiliation. Serum CST levels were determined by enzyme-linked immunosorbent assay (ELISA), using a commercially-available diagnostic kit (EK-053-27CE, EIA kit, Phoenix Pharmaceuticals Inc., Burlingame, CA, USA). According to the manufacturer’s instruction, the reported sensitivity of the assay kit for CST was 0.05 ng/mL with a linear range of 0.05–0.92 ng/mL. Cross-reactivity with endogenous human CST peptide for this assay kit was 100% (manufacturer declared intra-assay and inter-assay coefficients of variation were < 10% and < 15%, respectively). CRP and rheumatoid factor (RF) were determined by the immunoturbidimetric method (Roche^®^ Diagnostics GmbH, Mannheim, Germany). Anti-citrullinated protein antibodies (ACPA) were determined by chemiluminescence microparticle immunoassay on an Architect analyzer (Abbott, Abbott Park, IL, USA). Other biochemical analyses were conducted using standard laboratory procedures.

### Assessment of disease activity

DAS score in RA patients was performed by two experienced rheumatology specialists independently and score was estimated using the online Dawn^®^ visual DAS28 calculator^30^, using number of tender and swollen joints, levels of an acute phase reactant [either ESR (mm/h) or CRP (mg/L)] plus measure of general health [GH; patient assessment of disease activity using a 100 mm visual analogue scale (VAS) with 0 = best, 100 = worst]. Disease activity was graded according to the following thresholds: DAS-28 score < 2.6 indicates remission; score from 2.6 to 3.2 indicates low disease activity; score from 3.2 to 5.1 indicates moderate disease activity and; DAS-28 score above 5.1 is considered high disease activity^31^.

### Measurement of functional disability

The HAQ was used to examine the extent of functional ability in daily living and activities in patients with RA. The HAQ score ranges from 0 to 3 and is a good predictor of future disability. HAQ was performed by an experienced rheumatology specialist. The HAQ evaluates the patient’s level of functional ability through 20 questions in eight categories, which include the patient’s disturbance of movements and activities that involve both upper and lower extremities. In accordance with previous findings, a score below 0.5 is considered normal, whereas a score above 1.5 indicates severe disability^32^.

### Statistical analysis

The data for the present study were analyzed using SPSS Statistics for Windows^®^ (version 25.0, IBM, Armonk, NY, USA) and MedCalc (MedCalc Software, Ostend, Belgium, version 19.1.2). Continuous data are presented as the mean ± standard deviation or median (interquartile range) based on the variable distribution normality, whereas categorical data are expressed as numbers (N) with percentages (%). Normality of distribution for continuous variables was assessed using the Kolmogorov–Smirnov test. For differences between the groups, an independent samples t-test was used for continuous variables with normal distribution, whereas the Mann–Whitney U test was employed for the analysis of continuous variables with non-normal distribution. The chi-squared (χ^2^) test was used to determine differences between groups in terms of categorical variables. To investigate the correlation between CST and various clinical parameters, we used either Pearson’s correlation coefficient or Spearman’s rank correlation coefficient based on the variable distribution normality. Comparison of serum CST levels between RA duration tertiles was performed using one-way analysis of variance (ANOVA) with a post hoc Tukey test. Finally, a multiple linear regression analysis with a forward algorithm was applied to determine significant and independent correlates of the CST serum levels, which was defined as a dependent continuous variable. The level of statistical significance was set at a *p*-value < 0.05.

### Sample size analysis

Sample size analysis was conducted using the data from a pilot study on 10 subjects from the RA population and 10 matched control subjects. The value of serum CST, which was the main result of the study, was used for the calculation. The mean serum CST levels were 9.40 ± 2.75 ng/mL in the RA group and 3.71 ± 2.55 ng/mL in the control group. With a type I error of 0.05 and a power of 90%, the required sample size was 29 participants per group.

## Results

### Baseline characteristics and laboratory parameters

There were no statistically significant differences in anthropometric measurements between the RA patients and the healthy control group. The demographic and anthropometric characteristics of the RA patients and the healthy control group are shown in Table 1. The median duration of RA was 15.0 (10.0–20.0) years, while 51 (63.7%) patients had a positive RF and 56 (70%) had a positive ACPA (Table 1).

**Table 1:** Baseline characteristics of the RA group and the control group.

Parameter	RA group (N = 80)	Control group (N = 80)	*p*
Female sex (N, %)	72 (90.0)	70 (87.5)	0.802*
Age (years)	56.1 ± 12.2	53.0 ± 13.5	0.126**
Body weight (kg)	73.2 ± 13.6	70.0 ± 14.0	0.145**
Body height (cm)	169.3 ± 7.1	168.2 ± 9.8	0.441**
Body mass index (kg/m^2^)	25.5 ± 4.1	24.6 ± 4.0	0.163**
SBP (mmHg)	131.0 ± 17.5	126.8 ± 15.6	0.113**
DBP (mmHg)	80.3 ± 10.7	78.1 ± 11.1	0.209**
Smoking (N, %)	27 (34.2)	23 (28.7)	0.571*
Disease duration (years)^†^	15.0 (10.0–20.0)	–	–
Rheumatoid factor (N, %)	51 (63.7)	–	–
Anti-citrullinated protein antibodies (N, %)	56 (70)	–	–
DAS28 (score)	2.52 ± 0.96	–	–
HAQ (score)	0.84 ± 0.59	–	–
csDMARD	55 (68.7)	–	–
tsDMARD	12 (15)	–	–
bDMARD	68 (83.7)	–	–

Data are presented as the whole number (percentage), mean ± standard deviation or median (IQR).

*SBP* systolic blood pressure, *DBP* diastolic blood pressure, *DAS28* Disease Activity Score 28, *HAQ* Health Assessment Questionnaire, *csDMARD* conventional synthetic disease-modifying antirheumatic drug, *tsDMARD* targeted synthetic disease-modifying antirheumatic drug, *bDMARD* biologic disease-modifying antirheumatic drug.

*Chi-square test.

**t-test for independent samples.

^†^Time period since the initial diagnosis.

In the laboratory analyses, the RA group had significantly higher levels of serum creatinine (68.9 ± 16.9 vs 64.0 ± 11.1 μmol/L, p = 0.030), hsCRP (3.6 ± 2.6 vs 1.2 ± 1.1 mg/L, p < 0.001), total cholesterol (5.3 ± 1.1 vs 4.8 ± 1.0 mmol/L, p = 0.007) and LDL (3.2 (2.5–3.7) vs 2.8 (2.1–3.6) mmol/L, p = 0.034). Moreover, the RA group had a significantly lower level of 25OHD (39.5 ± 5.1 vs 43.7 ± 2.4 nmol/L, p < 0.001) (Table 2).

**Table 2:** Laboratory parameters of the RA group and the control group.

Parameter	RA group (N = 80)	Control group (N = 80)	*p*
Erythrocytes (× 10^12^/L)	4.4 ± 0.4	4.5 ± 0.4	0.275**
Hemoglobin (g/L)	133.8 ± 13.0	136.3 ± 10.7	0.192**
Fasting glucose (mmol/L)	4.9 ± 0.6	4.7 ± 0.5	0.143**
TSH (mIU/mL)	2.2 (1.3–3.2)	1.7 (1.2–3.0)	0.334***
25OHD (nmol/L)	39.5 ± 5.1	43.7 ± 2.4	<0.001**
Urea (mmol/L)	5.4 ± 1.7	5.2 ± 1.6	0.513**
Creatinine (μmol/L)	68.9 ± 16.9	64.0 ± 11.1	0.030**
eGFR (mL/min/1.73 m^2^)	83.0 (70.4–96.2)	86.3 (75.2–102.7)	0.164****
hsCRP (mg/L)	3.6 ± 2.6	1.2 ± 1.1	<0.001**
Triglycerides (mmol/L)	1.4 ± 0.7	1.3 ± 0.6	0.552**
Total cholesterol (mmol/L)	5.3 ± 1.1	4.8 ± 1.0	0.007**
HDL cholesterol (mmol/L)	1.7 ± 0.4	1.8 ± 0.5	0.363**
LDL cholesterol (mmol/L)	3.2 (2.5–3.7)	2.8 (2.1–3.6)	0.034***

Data are presented as the mean ± standard deviation and median (IQR).

*TSH* thyroid stimulating hormone, *25OHD* 25 hydroxyvitamin D, *eGFR* estimated glomerular filtration rate, *hsCRP* high sensitivity C-reactive protein.

**t-test for independent samples.

***Mann–Whitney U test.

****Calculated by MDRD equation.

### Serum catestatin levels

**Figure 1:**
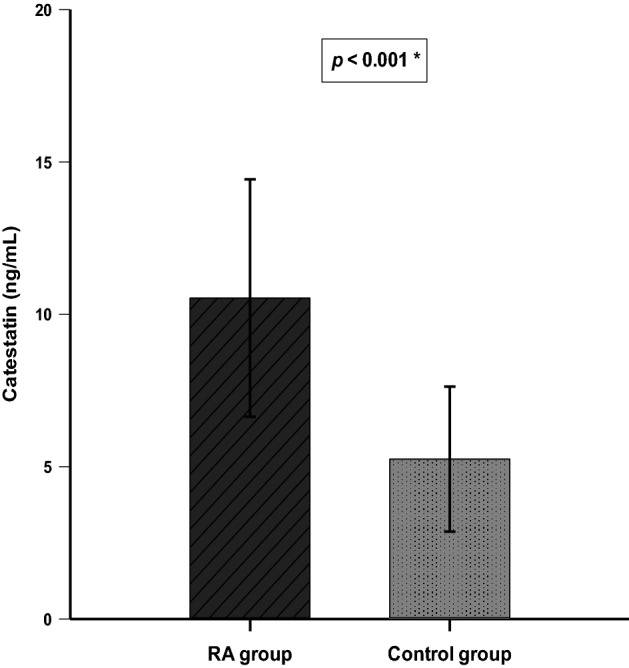
Comparison of serum catestatin levels between the RA group (N = 80) and the control group (N = 80). *t-test for independent samples.

Serum CST levels were significantly higher in patients with RA than in the control group (10.53 ± 3.90 vs 5.24 ± 2.37 ng/mL, p < 0.001) (Fig. 1).

### Catestatin correlations with laboratory and anthropometric parameters in the RA group

In patients with RA, there was a statistically significant correlation between CST levels and age (r = 0.418, *p* < 0.001). There were no other statistically significant correlations between catestatin levels and laboratory or anthropometric parameters (Table 3).

**Table 3:** Correlation analysis between serum catestatin levels and biochemical and anthropometric parameters in the RA group.

Parameter	r*	*p*
hsCRP (mg/L)	0.163	0.150
Fasting glucose (mmol/L)	0.065	0.568
Triglycerides (mmol/L)	0.114	0.315
Total cholesterol (mmol/L)	0.155	0.173
HDL (mmol/L)	0.126	0.266
LDL (mmol/L)	0.130^‡^	0.252
Urea (mmol/L)	0.164	0.148
Creatinine (μmol/L)	0.115	0.313
25OHD (nmol/L)	0.020	0.860
TSH (mIU/mL)	0.045^‡^	0.696
Age (years)	0.418	< 0.001
Body mass index (kg/m^2^)	0.098	0.391
SBP (mmHg)	0.133	0.241
DBP (mmHg)	0.131	0.248

*hsCRP* high sensitivity C-reactive protein, *25OHD* 25 hydroxyvitamin D, *TSH* thyroid stimulating hormone, *SBP* systolic blood pressure, *DBP* diastolic blood pressure.

*Pearson’s correlation coefficient, N = 80.

^‡^Spearman’s rank correlation coefficient.

### Catestatin correlation with RA activity scores and disease duration

**Figure 2:**
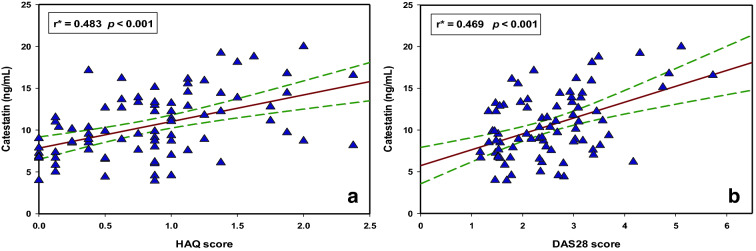
Correlation between serum catestatin levels and (**a**) HAQ score and (**b**) DAS28 score (N = 80). *Pearson’s correlation coefficient.

There was a significant positive correlation of serum CST levels with both DAS28 (r = 0.469, *p* < 0.001) and HAQ score (r = 0.483, *p* < 0.001) (Fig. 2).

**Figure 3:**
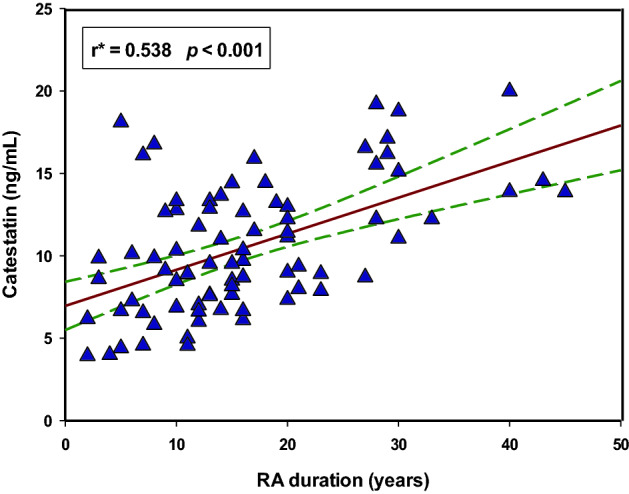
Correlation between serum catestatin levels and RA duration (N = 80). *Spearman’s correlation coefficient.

Moreover, there was a statistically significant positive correlation between serum CST levels and RA duration (r = 0.583, *p* < 0.001) (Fig. 3).

### CST and RA duration

The RA group was divided into tertiles depending on the disease duration. The 1st tertile was 2–11 years; the 2nd was 12–17 years, and the 3rd was > 17 years of duration.

**Figure 4:**
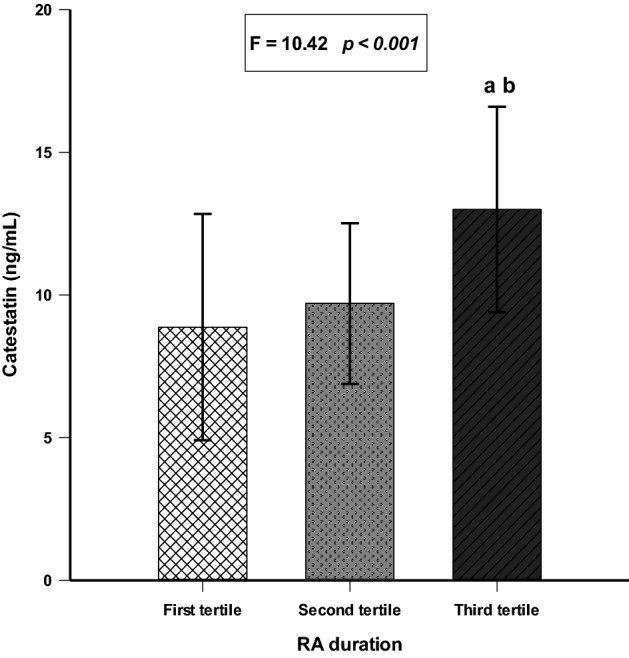
Comparison of serum catestatin levels between RA duration tertiles. *First tertile N = 27; Second tertile N = 26; Third tertile N = 27. One-way analysis of variance (ANOVA) with a post hoc Tukey test was used to examine differences between each of the groups. ^a^*p* < 0.05 *vs* first RA duration tertile. ^b^*p* < 0.05 *vs* second RA duration tertile.

Comparison of serum CST levels between RA duration tertiles (F-ratio = 10.42, *p* < 0.001) showed significantly higher levels of serum CST in the 3rd tertile (12.99 ± 3.60 ng/mL) than in both the 2nd (9.70 ± 2.81 ng/mL) and 1st (8.87 ± 3.97 ng/mL) tertiles (*p* < 0.05) (Fig. 4).

### Multiple linear regression analysis

Multiple linear regression analysis showed that serum CST levels retained a significant association with RA duration (β ± SE, 0.13 ± 0.04, *p* = 0.002) and DAS28 (0.94 ± 0.45, *p* = 0.039) after model adjustment for age, BMI and HAQ score, with serum CST levels as a dependent variable (Table 4).

**Table 4:** Multiple linear regression model of independent predictors for serum catestatin levels.

Variable	β^†^	SE^‡^	t-value	*p*
Age (years)	0.041	0.035	1.167	0.247
Body mass index (kg/m^2^)	0.051	0.089	0.580	0.563
DAS28 (score)	0.943	0.450	2.094	0.039
HAQ (score)	1.201	0.745	1.611	0.111
Disease duration (years)	0.133	0.042	3.144	0.002

*DAS28* Disease Activity Score 28; *HAQ* Health Assessment Questionnaire.

^†^Unstandardized coefficient β.

^‡^Standard error.

## Discussion

This study showed that patients with RA who were treated with either bDMARDs or tsDMARDs had significantly higher levels of serum CST than healthy control subjects. Furthermore, CST had a significant positive correlation with both DAS28 and HAQ scores, as well as with RA duration. To the best of our knowledge, this is the first clinical study that evaluates the serum concentration of CST in patients with RA and its correlation with specific disease characteristics.

As already mentioned, CST participates in the regulation of hypertension and cardiac functions and in promoting angiogenesis^13,33^. The data from several experimental models and in vivo studies indicated that high plasma concentrations of CgA in RA patients could be involved in persistent vascular inflammation, endothelial barrier dysfunction and systemic sympathetic hyperactivity^26,34,35^. Moreover, it was proposed that noticeable increase in CgA levels in RA, potentially could induce the attenuation of its anti-angiogenic impact^36^. Accordingly, plasma CgA could be used as both a diagnostic and prognostic marker in various inflammatory conditions, including RA^27,34^. Based on these findings, elevated levels of the CgA degradation product CST in RA patients could be expected, which was confirmed by this study. Our results imply that CST plays a role in the pathophysiology of RA, which is consistent with its well-established systemic anti-inflammatory effects^13,27,37–39^.

In addition to higher CST levels in our group of RA patients than in healthy subjects, we found that CST is an independent predictor of disease activity and functional disability. Based on the existing literature, a possible explanation for these findings is that CST participates in a cascade of signal transduction pathways between immune and noimmune cells, particularly altering the balance between pro- and anti-inflammatory cytokines^39^. Furthermore, these results would presume that a more active disease triggers regulatory mechanisms that lead to compensatory secretion of CST to protect the host from the chronic inflammatory process. Experimental animal studies in colitis and atherosclerosis mouse models have shown that administration of CST reduces the prevalence of macrophages and monocytes in inflamed tissues^13,40^. There is an abundance of macrophages in the inflamed synovial membrane that strongly promote overexpression of proinflammatory cytokines^41,42^. CST could attenuate the intensity of inflammation in RA through modification of the differentiation of monocytes to macrophages. Although the mechanisms by which CST affects monocyte and macrophage migration are still unclear, there are several assumptions about this effect. One possible pathophysiological mechanism includes the reductive effect of CST on the expression of vascular cell adhesion molecule-1 (VCAM-1) and consequent changes in the inflammatory environment^13^. VCAM-1 is a glycoprotein expressed by endothelial cells that mediates leukocyte-endothelial cell adhesion during inflammation^41^. Several studies reported significantly higher serum VCAM-1 levels in patients with RA^42–44^. In addition, serum VCAM-1 levels correlate with endothelial dysfunction and the initiation and progression of RA^42,43,45^. The expression of VCAM-1 in synovial tissue is upregulated by proinflammatory cytokines (TNF-α and IL-6)^45^. By lowering the production of proinflammatory cytokines, CST could decrease the expression of adhesion molecules in the endothelium. Moreover, an in vitro study showed that anti-VCAM-1 monoclonal antibody injection into a collagen-induced RA mouse model did not alter the incidence, but decreased the severity of arthritic joints compared with the control mice^46^. Another possible mechanism by which CST regulates macrophages refers to the polarization of RA synovial tissue “activated macrophages” towards two different phenotypes depending on the disease activity: M1 (classically activated) and M2 (alternatively activated)^47^. The M1 phenotype releases high levels of proinflammatory cytokines and consequently prevails in patients with highly active RA alongside the increased presence of proinflammatory macrophage receptor with collagenous domain (MARCO)^48^. Based on the results of in vivo and in vitro studies, CST treatment decreases proinflammatory cytokine (IL-6, IL-1β, TNF-α) and macrophage levels^13,17,40^. Additionally, the production of CST by macrophages could result in reduction of the inflammation by feedback inhibition mode^15^. Considering that the RA population investigated in this study was treated with biological agents, a synergistic effect of those drugs and CST in RA is possible. Although this could, to some extent, explain the association between CST and RA, further studies are necessary to confirm the precise role of CST in the complex pathophysiology of RA.

Limited data indicate that high CST levels could reflect advanced CV disease burden, a burden that is significantly increased in RA^49^. Moreover, CVDs are still the leading cause of mortality in patients with RA (30–40% of deaths)^4,5,50,51^. Additionally, it has been established that both traditional CV risk factors and intrinsic RA features contribute to excess CV-related morbidity and mortality^22^. The most significant intrinsic RA features are the disease duration, the presence of ACPAs, RF, elevated proinflammatory cytokines and hsCRP serum levels^52–57^. Premature atherosclerosis is the hallmark of CVD development in RA^3^. According to this point, the precise effects of CST on atherosclerosis have not yet been clarified. A recent animal study conducted on apolipoprotein-deficient mice that were fed a high cholesterol diet showed that administration of CST to the same mice significantly reduced aortic atherosclerotic lesions and attenuated macrophages. Furthermore, by in vitro analysis, the authors determined that CST could suppress atherogenesis by inhibiting inflammatory responses in endothelial cells and macrophages^13^. In addition, the administration of CST to the mouse hindlimb ischemia model resulted in improvement of blood flow and reduction of necrosis^38^. Given these considerations, we hypothesize that the effects of CST on the inflammatory process in patients with RA could interfere with the process of atherogenesis and CV risks themselves. However, all of these findings should be interpreted with caution and need to be explored in further, large-scale studies.

This study has several limitations. First, our study was designed as a cross-sectional study, thereby preventing the establishment of causal relationships. Second, it had single-centre patient inclusion with a relatively small sample size. Additionally, due to technical restrictions, we were not able to measure all inflammatory parameters, which could potentially have an impact on some of the results.

In conclusion, this study showed that patients with RA have higher serum CST levels than healthy controls. Furthermore, CST was positively correlated with HAQ and DAS28 scores and disease duration. These findings imply that CST possibly plays a role in RA complex pathophysiology, especially in the severity of inflammation and atherogenesis. However, future larger multicentre longitudinal studies are necessary to explain the association between CST and RA.

## Data Availability

The datasets used and/or analysed during the current study are available from the corresponding author on reasonable request.
